# TCF7L2 regulates pancreatic β-cell function through PI3K/AKT signal pathway

**DOI:** 10.1186/s13098-019-0449-3

**Published:** 2019-07-05

**Authors:** Hui-Hui Wu, Yan-Liang Li, Nai-Jia Liu, Zhen Yang, Xiao-Ming Tao, Yan-Ping Du, Xuan-Chun Wang, Bin Lu, Zhao-Yun Zhang, Ren-Ming Hu, Jie Wen

**Affiliations:** 1 Department of Endocrinology and Metabolism, Jing’an District Center Hospital of Shanghai, Shanghai, 200040 China; 2 Department of Endocrinology and Metabolism, Huashan Hospital of Fudan University, NO. 12 Wulumuqi Mid Road, Building 0#, Jing’an District, Shanghai, 200040 China; 3 Department of Endocrinology and Metabolism, Xin Hua Hospital, Shanghai Jiao Tong University, Shanghai, 200020 China; 4 Department of Endocrinology and Metabolism, Hua Dong Hospital, Fudan University, Shanghai, 200040 China

**Keywords:** TCF7L2, PI3K/AKT signal pathway, Insulin secretion, PIK3R1

## Abstract

**Background:**

Transcription factor 7-like 2 (TCF7L2), which previously known as TCF-4, is a major form of transcription factor involved in the downstream WNT signaling and exhibits the strongest association to diabetes susceptibility. Although we still do not know mechanistically how TCF7L2 exerts its physiological functions on pancreatic endocrine cells, it had been suggested that TCF7L2 may directly affect β-cell function by regulating the activation of PI3K/AKT signaling pathway.

**Methods:**

MIN6 cells were transfected with TCF7L2 knockdown virus or lenti-TCF7L2 virus for 48 h to evaluate the contribution of TCF7L2 to the PI3K/AKT signaling pathway and pancreatic β-cell function. This was confirmed by measuring the expression of PI3K p85 and p-Akt by western blotting and insulin secretion by enzyme-linked immunosorbent assay (ELISA), respectively. Chromatin immunoprecipitation (ChIP) and polymerase chain reaction (PCR) experiments were performed to explore the genomic distribution of TCF7L2-binding sites in the promoter of PIK3R1, the affinity between which was analyzed by the luciferase reporter assay.

**Results:**

In the present study, we strikingly identified that TCF7L2 could profoundly inhibit the expression of PIK3R1 gene and its encoding protein PI3K p85, which then could lead to the activation of PI3K/AKT signaling and stimulate insulin secretion in pancreatic β-cells. However, the integrity and stability of evolutionarily conserved TCF7L2-binding motif plays a very crucial role in the binding events between transcription factor TCF7L2 and its candidate target genes. We also found that the affinity of TCF7L2 to the promoter region of PIK3R1 alters upon the specific binding sites, which further provides statistical validation to the necessity of TCF7L2-binding motif.

**Conclusions:**

This study demonstrated that TCF7L2 is closely bound to the specific binding regions of PIK3R1 promoter and prominently controls the transcription of its encoding protein p85, which further affects the activation of PI3K/AKT signaling pathway and insulin secretion.

## Background

Common variation within the gene encoding transcription factor 7-like 2 (TCF7L2) is now considered to be definitively associated with diabetes susceptibility. Despite that this association was identified in 2006 [1] and has been readily replicated in populations of different ethnic descent [2–5], the mechanism through which TCF7L2 exerts its effect on type 2 diabetes mellitus (T2DM) is still very unclear.

TCF7L2 (previously known as TCF-4) is a high-mobility group box-containing transcription factor and operates at the end point of the canonical Wnt signaling transduction cascade. During Wnt activation, TCF7L2 was activated by Wnt ligands or certain growth factors (such as insulin and IGF-1), and further participated in many Wnt-related biological processes [6]. Previous study suggested that TCF7L2 may stimulate the proliferation of pancreatic β-cells and facilitate the production of the incretin hormone glucagon-like peptide-1 in intestinal endocrine cells [7–9], but the molecular mechanisms underlying are far from understood. TCF7L2 also plays an important role in conveying Wnt signaling pathway in regulating gene expression, and a highly significant proportion of genes bound by TCF7L2 are known disease-associated loci [10, 11]. Chromatin immunoprecipitation (ChIP) has been taken directly to identify the DNA sequences bound by transcription factors in vivo [12]. As an experimentally generated list of potential transcription factor target genes has been reported in colorectal cancer cells using ChIP-on-Chip analysis [13], previous studies have also identified a serious of potential target genes of TCF7L2 (including MafA, Isl1, Pdx1, Axin2, PC1, PC2, and CPE), despite that the mechanistic data is still lacking [14–16].

Phosphoinositide 3-kinase (PI3K)/protein kinase B (AKT) pathway, another downstream target of Wnt signaling pathway, has been put in a well-established role in providing proliferative signal and participating in energetic metabolism [17, 18]. Recent data has shown that TCF7L2 could activate PI3K/AKT pathway in numerous diseases, such as prostate cancer and T2DM [19], which might also explain why TCF7L2 confers so many functions in the incidence and development of metabolic diseases. The PI3K/AKT pathway is the primary pathway of insulin signaling transduction, through which insulin regulates blood glucose balance [20]. PI3K consists of a catalytic subunit (p110) and a regulatory subunit (p85). The p85 subunit provides stability and maintains the activity of the p85/p110 complex. Reduced expression or functional defects of each subunit may lead to glucose metabolism disorder [21, 22]. Several studies have shown a beneficial role of PIK3R1 (the gene encoding p85 protein of PI3K) in the regulation of glucose trafficking and utilization [23]. So we hypothesized that TCF7L2 might modulate the PI3K/AKT signaling via targeting PIK3R1, but the concrete interaction remains unclear.

As a transcriptional regulator of Wnt signaling pathway, TCF7L2 may act as either a stimulator or a repressor of gene expression due to differential splicing and interaction with different co-regulators in target gene recognition [24]. It is well established that the various genes that are bound and transcriptionally activated by TCF7L2 differ markedly between cell types [25]. This is not entirely surprising, as it is clear that TCF7L2 is benignly bound to many regions in the genome. However, the binding-site specificity of TCF7L2 still needs to be determined. Using different in vitro approaches, we defined the optimal TCF7L2-binding motif as evolutionarily conserved TCAAAG motifs, the validity of which was further demonstrated and underscored in various species [26, 27]. Most of the TCF7L2 binding sites are distributed in clusters surrounding putative target genes [10, 28], and the sequences TCF7L2-binding motif contained were more conserved compared to random genomic segments, as expected for functional transcriptional regulatory regions [26]. The stability of TCF7L2-binding motif is undoubtedly indispensable for TCF7L2 to regulate the promoter activities of its candidate target genes [27].

The principal aims of this study were to determine the impacts of altering the expression level of TCF7L2 on insulin secretion and changes of PI3K/AKT signaling pathways activated by glucose in pancreatic β-cells. Then we destroyed the integrity of TCF7L2-binding motif by mutating the nucleotides CAA to make a concrete analysis of the binding events between TCF7L2 and PIK3R1 promoter at transcriptional level.

## Methods

### Cell lines and antibody

Mouse pancreatic islet beta-cell line of MIN6 and human embryonic kidney cell line-HEK293T were cultured at 37 °C and 95% humidity, and supplied with 5% CO_2_ in Dulbecco’s modified Eagle’s medium (DMEM) supplemented with 10% fetal bovine serum (Life Technologies, Carlsbad, CA), 100 µg/mL penicillin–streptomycin, 100 µg/mL l-glutamine, 0.05 µg/mL β-mercaptoethanol (Gibco Invitrogen, Carlsbad, CA, USA). After 65% cells attached, we synchronized them by the serum-free medium for 8 h. Insulin concentrations were measured in supernatants of incubation experiments via enzyme-linked immunosorbent assay. The antibody used in this study: TCF7L2 (Cell signaling Technology, USA).

### Cell transfection

TCF7L2 knockdown virus (KD1, KD3) or lenti-TCF7L2 were delivered at a final concentration of 50 nM using Lipofectamin 3000™ according to manufacturer’s instructions (Thermo Fisher Scientific). MIN6 cells were plated on 6-well plates at 5 × 10^5^ cells/well in triplicate for each transfection condition. After 24 h and 60–70% confluence, the cells in each well were transfected. After incubation for 12 h at 37 °C under 5% CO_2_, the medium was replaced with DMEM, with 15% heat-inactivated fetal bovine serum for another 48 h. After transfection, green fluorescence was used to evaluate transduction efficiency. TCF7L2 knockdown efficiency was assessed by western blot.

### Western blots

Cells were harvested with RIPA lysis buffer (Beyotime Inc, China) and centrifuged at 12,000 rpm for 10 min at 4 °C to collect the supernatant. 5 × Sodium dodecyl sulfate (SDS) loading buffer was added to the supernatant before denaturation at 100 °C for 10 min. Protein concentrations were determined by A BCA kit (Beyotime Inc, China). The protein extracts were separated by SDS-polyacrylamide gel electrophoresis (PAGE) to polyvinylidene difluoride (PVDF) membranes and transferred to PVDF membranes. After blocked in Tris-buffered saline containing 0.05% Triton X-100 (TBS-T) and 4% milk for 2 h, proteins were probed with primary antibodies overnight at 4 °C. The proteins were visualized by ECL chemiluminescence. Density of the bands was analyzed by using Lab Work 45 Image Software. Each experiment was performed at least three times.

### Chromatin immunoprecipitation (ChIP)

ChIP was performed in triplicate following the instructions provided by the suppliers of the Pierce Agarose ChIP Ki (Thermo). Briefly, MIN6 cells were cross-linked with 1% formaldehyde for 20 min at room temperature and sonicated on ice for 15 cycles of 30 s on and 30 s off (Bioruptor UCD-200; Diagenode, Liège, Belgium). The sonicated chromatin was primarily in the 200 to 1000 bp range, which was successively centrifuged for 15 min and incubated overnight at 4 °C with either Anti-TCF7L2 (Cell Signaling Technology, USA) and non-immune IgG (Santa Cruz Biotechnologies, USA) for negative control at 1 µg of antibody per 10^6^ cells. The precipitated chromatin was eluted at room temperature for 20 min, de-cross-linked by incubation at 65 °C for 5 h in the presence of 200 mM NaCl, extracted with phenol–chloroform, and precipitated.

### Quantitative polymerase chain reaction (PCR) analysis

ChIP experiments were analyzed with quantitative PCR (34 cycles of denaturation at 95 °C for 2 min, annealing at 55 °C for 30 s and extension at 72 °C for 30 s) by using Applied Biosystems 7000 Real-Time PCR System (Applied Biosystems, Thermo, United States). ChIP values were normalized as a percentage of Input. The results of the RT-PCR analysis were determined based on the threshold cycle (Ct), and the relative expression levels were calculated using the 2^−ΔΔCT^ method. All reactions were run in triplicate.

### Luciferase reporter assay

For luciferase reporter constructs, the pGL3 was used as the vector backbone. Human PIK3R1 promoter containing wild-type or mutated TCF7L2-binding sites was amplified and then inserted into the pGL3 basic vector to build luciferase plasmids. HEK293T cells were cultured in 96-well plates in the presence of medium (DMEM supplemented with 10% fetal bovine serum) and transfected with pGL3 constructs and Renilla luciferase expression vectors by using Lipofectamine^®^ 2000 (Invitrogen, Carlsbad, CA, USA). The luciferase activity was measured 48 h later using the Dual-luciferase reporter system of Spectra Max M5 instrument (Promega) according to the manufacturer’s protocol. Firefly luciferase activity was normalized to *Renilla* luciferase activity.

### Glucose stimulated insulin secretion (GSIS) in MIN6 cells in vitro

Starve MIN6 cells with DMEM (0.1% BSA and 2.8 mM glucose) overnight. Next, starve cells again with KRB buffer containing 125 mM NaCl, 4.74 mM KCl, 1 mM CaCl_2_, 1.2 mM KH_2_PO_4_, 1.2 mM MgSO_4_, 5 mM NaHCO_3_, 25 mM Hepes (pH 7.4) and 3 mM glucose for 1 h. The beads were successively washed 2 times with the same KRB buffer and then changed to KRB with either 2.8 mM or 16.7 mM glucose. Collect medium after 1 h, and then measure the insulin concentration using ELISA as described previously.

### Statistical analysis

For statistical evaluation and the significance testing of differences, the results are expressed as the mean ± SD (standard deviation). The nonparametric Mann–Whitney U test was performed with Graph Pad software (version 6.0c; Graph Pad Software, Inc., La Jolla, CA, USA). Statistical significance was set at *p* < 0.05. Each experiment was performed for more than 3 times.

## Results

### TCF7L2 positively regulates insulin secretion through PI3K/AKT signaling pathway

**Fig. 1:**
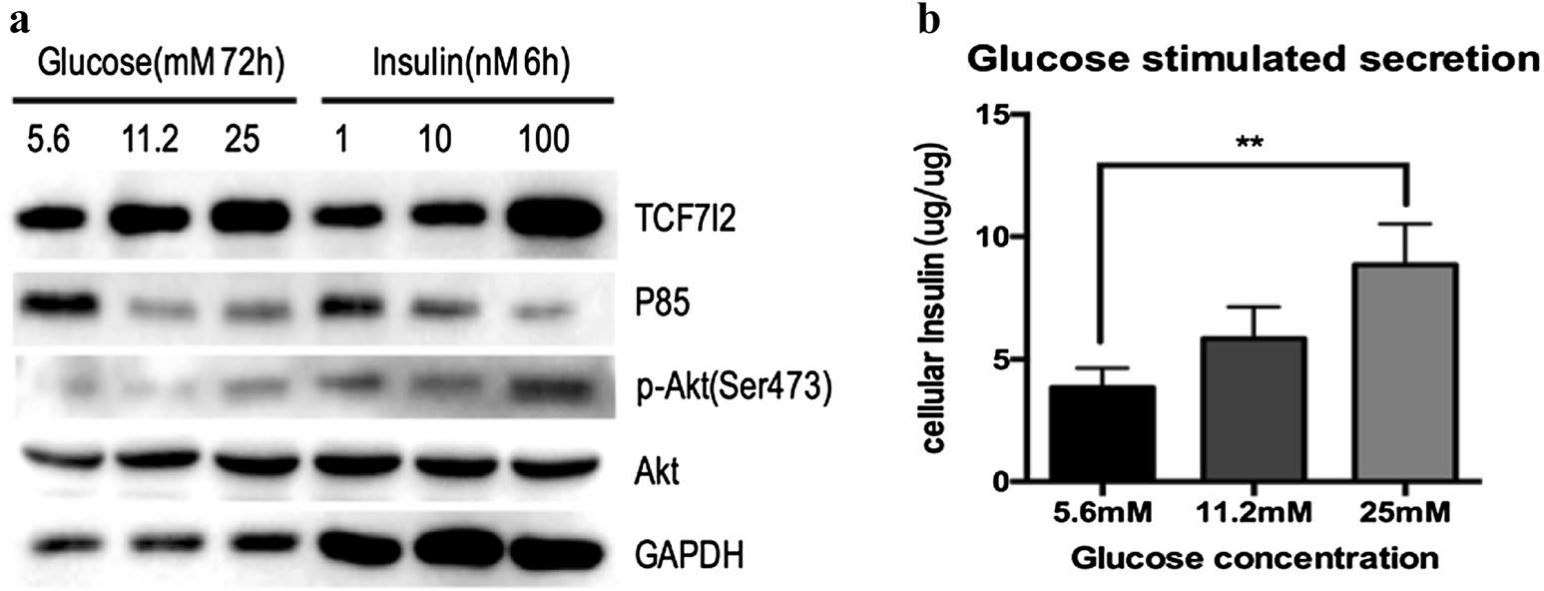
Effects of glucose or insulin on gene transcription and insulin secretion. MIN6 cells were cultured with 5.6 mM, 11.2 mM and 25 mM glucose for 72 h or with 1 nM, 10 nM, 100 nM insulin for 6 h. **a** The expressions of TCF7L2, p85, Akt were determined by western blotting. **b** GSIS was performed on MIN6 cells in vitro and insulin concentrations in the supernatants were measured using ELISA (n = 3 samples per group, **p < 0.01 as indicated)

MIN 6 were cultured with 5.6 mM, 11.2 mM and 25 mM glucose for 72 h or with 1 nM, 10 nM, 100 nM insulin for 6 h, after which TCF7L2, p85 and Akt expression were detected by western blotting (Fig. 1). With the increased concentrations of glucose or insulin in the culture of MIN6 cells, the expression of TCF7L2 increased and the gene PIK3R1 decreased, followed by the activation of AKT signaling.

**Fig. 2:**
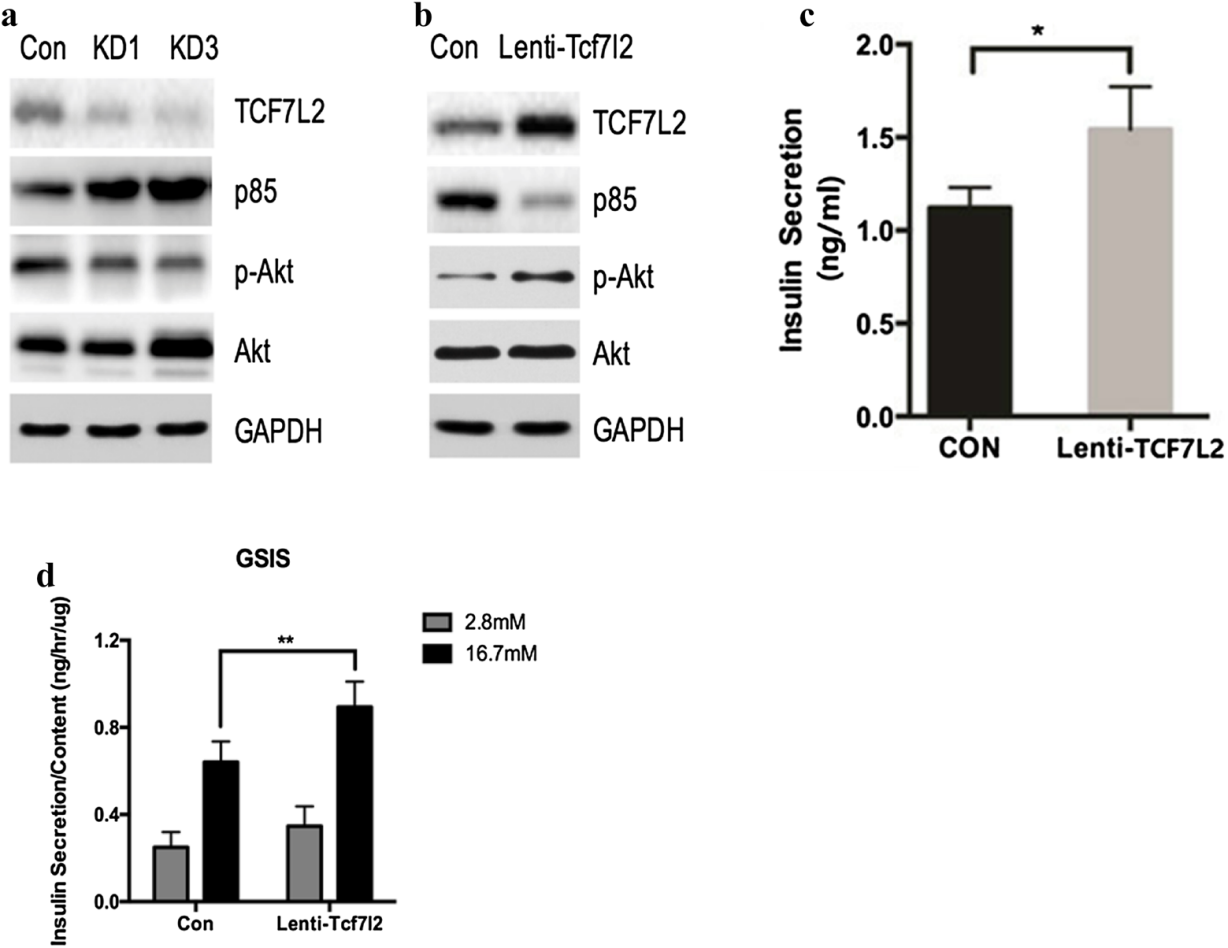
Impact of TCF7L2 on the expressions of TCF7L2, p85, Akt and insulin secretion. MIN6 cells were transfected with TCF7L2 knockdown virus (KD1, KD3) or lenti-TCF7L2 virus for 48 h. Then the expressions of TCF7L2, p85, Akt were evaluated by western blot (**a**, **b**) and insulin concentrations in the supernatants were measured using ELISA (**c**). GSIS was also performed on MIN6 cells in vitro to detect the effect of TCF7L2 over-expression on insulin secretion (**d**) (n = 3 samples per group, *p < 0.05 and **p < 0.01 as indicated. *p* phosphorylated, *Con* control)

As multiple previous observations have suggested, TCF7L2 might contribute to β cell proliferation and pancreatic β-cell function, we therefore examined whether down- or up-regulated of TCF7L2 impact insulin secretion in the present study. MIN6 cells were transfected with TCF7L2 knockdown virus (KD1, KD3) or lenti-TCF7L2 virus for 48 h, then the expression of TCF7L2, p85 and p-Akt were measured by western blotting and insulin secretion in supernatant was detected by ELISA (Fig. 2). As a result, knockdown of TCF7L2 promoted the expression of PIK3R1 and its encoding protein PI3K p85, as well as inhibited the activation of Akt. Over-expression of TCF7L2 inhibited the expression of p85, activated the AKT signaling pathway and stimulated insulin secretion (*p < 0.05, **p < 0.01).

### The binding events between TCF7L2 and PIK3R1 promoter

**Fig. 3:**
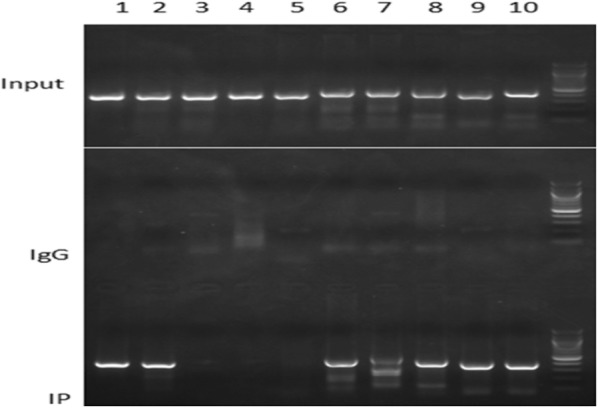
The ChIP-PCR products were separated by agarose gel electrophoresis

ChIP-PCR was utilized to identify and quantify the binding of TCF7L2 to PIK3R1. The promoter of PIK3R1 was divided into 10 segments and specific pairs of primers covering contiguous fragments of PIK3R1 promoter were designed and listed in Table 1. The detailed information of the subsections was also presented below. Chromatin quantified to 100 μg DNA was utilized for immunoprecipitation and diluted at a rate of 1:10 in IP buffer as the Input. For the identification of the DNA targets of TCF7L2, after immunoprecipitation of cross-linked chromatin, the DNA is purified and analyzed by agarose gel electrophoresis in mouse pancreatic islet beta-cell line of MIN6. This approach is rapid and sensitive and allows fine mapping of chromosomal proteins in regions as small as 300 bp. The IgG group represented negative control and the Input group served as positive control respectively. As shown in Fig. 3, the positive stripes about 500 bp appeared in the electrophoresis of the immunoprecipitated fractions presented a validated affinity of TCF7L2 to the specific segments of PIK3R1 promoter corresponding to primers 1–2 and 6–10. However, blank strips presented in the electrophoresis of specific sequences of PIK3R1 promoter which corresponding to primers 3 and 4, significantly due to the lack of TCF7L2 binding sites in the regions.

**Table 1:** PCR primers used to amplify contiguous fragments of PIK3R1 promoter

Primer	Forward	Reverse
1	5′-TTGTTCTGAGACAAAATAGAC-3′	5′-CCACTTCTTTTGAACCATTAA-3′
2	5′-TTAATGGTTCAAAAGAAGTGG-3′	5′-CAGTCAGCCCCGCAGGTGTTA-3′
3	5′-TAACACCTGCGGGGCTGACTG-3′	5′-GGGCAGGTTCAAATATTTGCT-3′
4	5′-AGCAAATATTTGAACCTGCCC-3′	5′-ACTTTGCAGGTTATGCATTTC-3′
5	5′-GAAATGCATAACCTGCAAAGT-3′	5′-CTCAGTAAAATCAGCCTAGCT-3′
6	5′-AGCTAGGCTGATTTTACTGAG-3′	5′-GGCTTTGTGGCTGTGAATGTT-3′
7	5′-AACATTCACAGCCACAAAGCC-3′	5′-TGCTCAATAGTGGTGAGTTTC-3′
8	5′-GAAACTCACCACTATTGAGCA-3′	5′-CGTGCATCTGGGTTTAGAGAT-3′
9	5′-ATCTCTAAACCCAGATGCACG-3′	5′-AGTCCGGCTTTCTTTGTAATG-3′
10	5′-TTATCAGCTCTCGTCAATCTGC-3′	5′-TGTGCGACAGTTTCCTTGGCT-3′

**Fig. 4:**
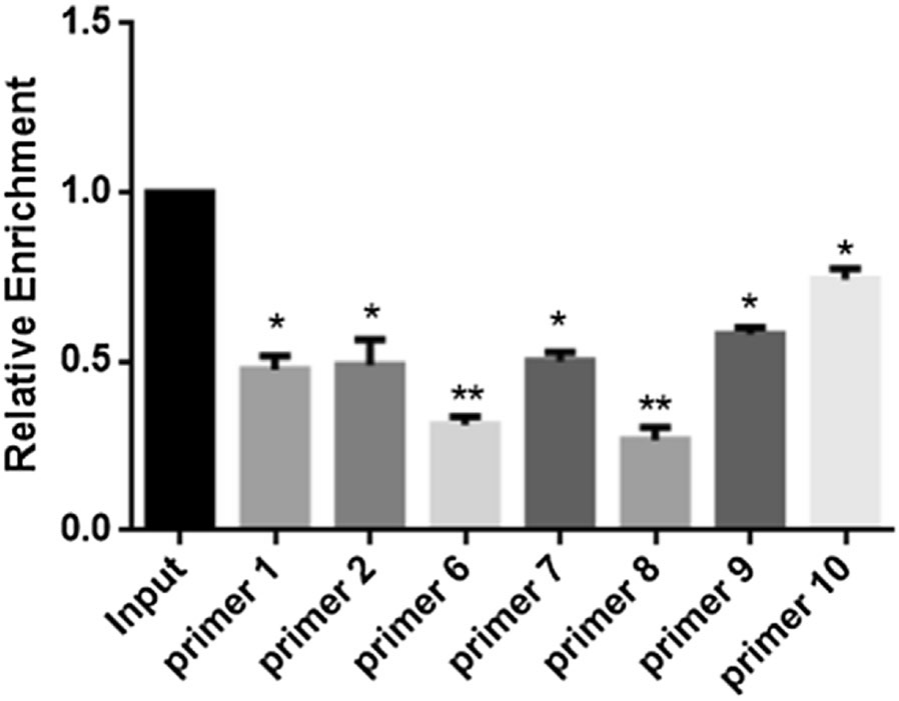
Relative enrichments of the immunoprecipitated fractions after RT-PCR (n = 3 samples per group, *p < 0.05, **p < 0.01 vs. input)

After DNA purification from immunoprecipitated chromatin, we selected the genomic fractions corresponding to specific protein-binding sites for real-time PCR. The relative enrichment of each group was presented and normalized as a percentage of Input. Statistic difference (p < 0.05) was observed between the experimental groups and the Input, suggesting that the binding strength of TCF7L2 varies upon the specific segments of PIK3R1 promoter (Fig. 4).
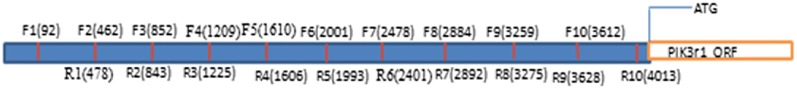

**Fig. 5:**
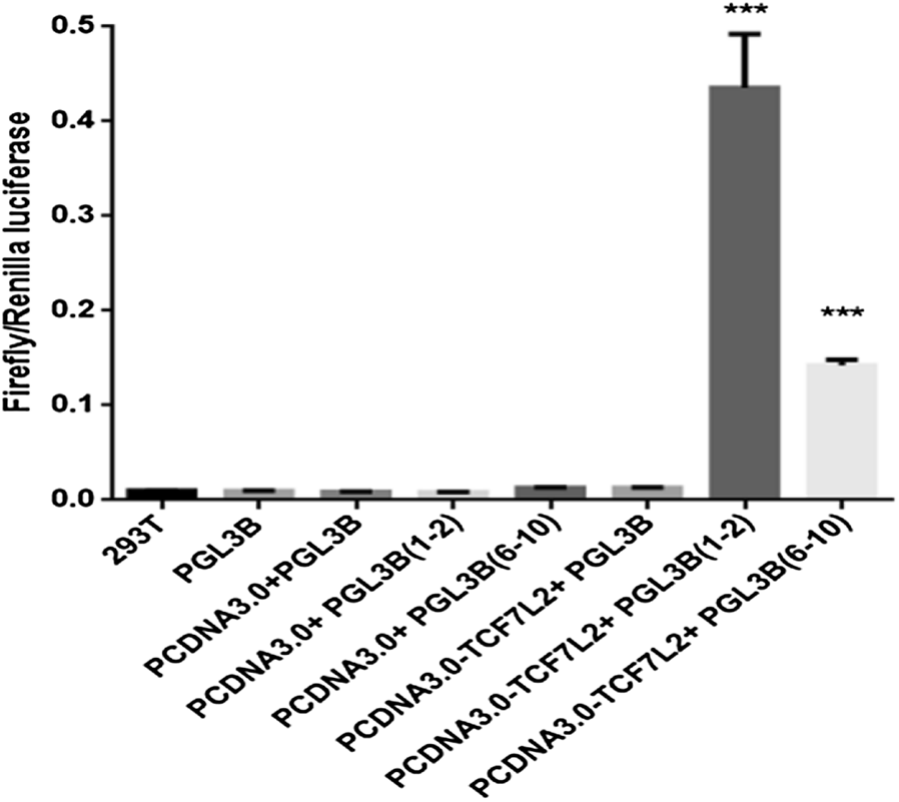
The detected samples were grouped into 8 groups depending on the plasmids that were tranfected, and the luciferase activity of each group was recorded. The 8 groups are: (i) 293T, (ii) PGL3B, (iii) PCDNA3.0 + PGL3B, (iv) PCDNA3.0 + PGL3B(1–2), (v) PCDNA3.0 + PGL3B(6–10), (vi) PCDNA3.0-TCF7L2 + PGL3B, (vii) PCDNA3.0-TCF7L2 + PGL3B(1–2), and (viii) PCDNA3.0-TCF7L2 + PGL3B(6–10) (*n *= 3 samples per group, ***p < 0.001 vs. PGL3B)

Next, luciferase reporter assay was applied to make further confirmation. In the analysis, TCF7L2-binding regions were cloned into the PGL3B vector and transiently transfected into HEK293T cells with cotransfection of the *Renilla* vector as the normalizing control. The detected samples were further subdivided into eight groups according to the plasmids they were tranfected. Values were expressed as activities relative to that of the empty PGL3B vector. Only peaks from group 7 and 8 were meaningful in the experiment, indicating that TCF7L2 could directly bind to the specific fragments of PIK3R1 promoter corresponding to primers 1–2 and 6–10. The relative higher luciferase activity represented the increased efficiently, which demonstrated that there was a striking bias for the binding of TCF7L2 to the DNA fragments of PIK3R1 promoter corresponding to primers 1–2 (Fig. 5).

### Distribution of TCF7L2-binding motif and its necessity in the binding events between TCF7L2 and PIK3R1 promoter

The previous defined optimal TCF7L2-binding motif was TCAAAG, which was confirmed by different approaches in vitro. To investigate the transcriptional regulatory activity of TCF7L2-bound regions, we located the position of TCF7L2-binding motif in the promoter region of PIK3R1. The location of TCF7L2-binding motif and primers used to amplify CAA-mutated fragments were shown in Table 2.

**Table 2:** The location of TCF7L2-binding motif in the promoter of PIK3R1 and primers used to amplify CAA-mutated fragments

Location of TCF7L2-binding motif	Forward	Reverse
381–384	5′-TTTTCCGTTAATGGTTAAGAAGTGGGGGCT-3′	5′-AGCCCCCACTTCTTAACCATTAACGGAAAA-3′
487–490	5′-TTAGCTGCAACTTTCTATTACAGACTAGAA-3′	5′-TTCTAGTCTGTAATAGAAAGTTGCAGCTAA-3′
1795–1798	5′-GCAGGAAGTTGGGGTGCCAGGGCTTGCAGG-3′	5′-CCTGCAAGCCCTGGCACCCCAACTTCCTGC-3′
1986–1989	5′-CTTTTGGGAGAAGACTCGTTTTAAATTAAA-3′	5′-TTTAATTTAAAACGAGTCTTCTCCCAAAAG-3′
2387–2390	5′-CCATCCATGTAAATGTGAGGTTCTGGCTCC-3′	5′-GGAGCCAGAACCTCACATTTACATGGATGG-3′
2420–2423	5′-CAGGGGCAACGTATATGCTCCAGTCTTGGT-3′	5′-ACCAAGACTGGAGCATATACGTTGCCCCTG-3′
3232–3235	5′-AGCTAGGCTGATTTTACTGAG-3′	5′-TTCAGCACAGGAATGAAGCTG-3′

**Fig. 6:**
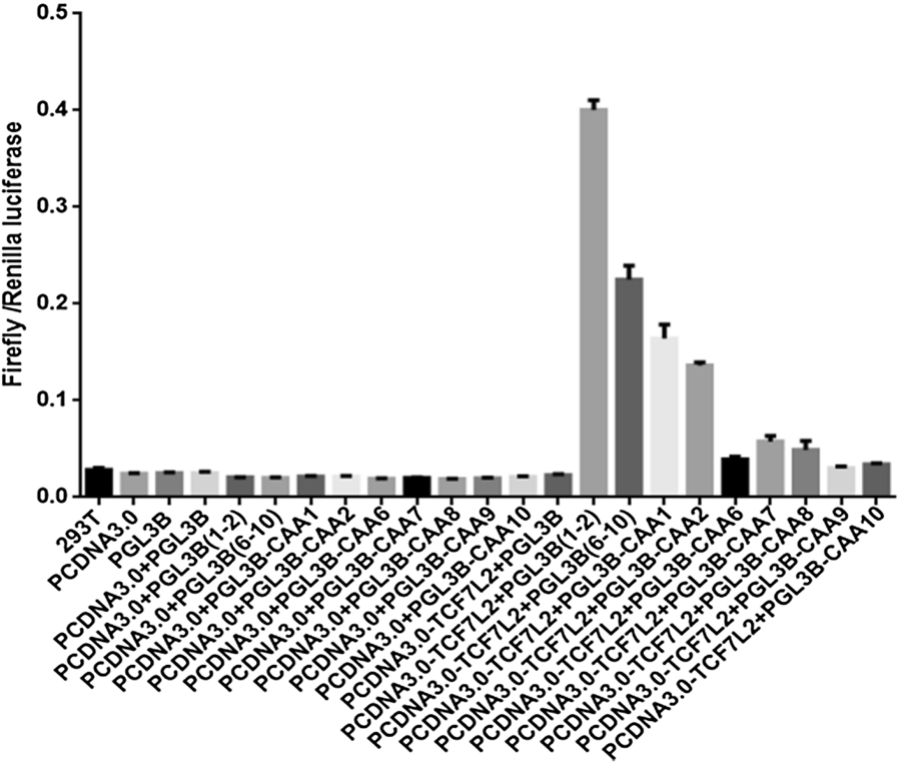
The luciferase activities of these 23 groups were recorded and analyzed. The 23 groups are: (i) 293T, (ii) PCDNA3.0, (iii) PGL3B, (iv) PCDNA3.0 + PGL3B, (v) PCDNA3.0 + PGL3B(1–2), (vi) PCDNA3.0 + PGL3B(6–10), (vii) PCDNA3.0 + PGL3B-CAA1, (viii) PCDNA3.0 + PGL3B-CAA2, (ix) PCDNA3.0 + PGL3B-CAA6, (x) PCDNA3.0 + PGL3B-CAA7, (xi) PCDNA3.0 + PGL3B-CAA8, (xii) PCDNA3.0 + PGL3B-CAA9, (xiii) PCDNA3.0 + PGL3B-CAA10, (xiv) PCDNA3.0-TCF7L2 + PGL3B, (xv) PCDNA3.0-TCF7L2 + PGL3B(1–2), (xvi) PCDNA3.0-TCF7L2 + PGL3B(6–10), (xvii) PCDNA3.0-TCF7L2 + PGL3B-CAA1, (xviii) PCDNA3.0-TCF7L2 + PGL3B-CAA2, (xix) PCDNA3.0-TCF7L2 + PGL3B-CAA6,(xx) PCDNA3.0-TCF7L2 + PGL3B-CAA7, (xxi) PCDNA3.0-TCF7L2 + PGL3B-CAA8, (xxii) PCDNA3.0-TCF7L2 + PGL3B-CAA9, and (xxiii) PCDNA3.0-TCF7L2 + PGL3B-CAA10 (*n *= 3 samples per group)

To further investigate whether TCF7L2-binding motif is necessary in the binding events between TCF7L2 and PIK3R1 promoter, mutated TCF7L2-binding sites was amplified and then inserted into the pGL3 empty vector to build luciferase plasmids, which were transfected into MIN6 cells afterwards. When the luciferase activities of groups carrying CAA-mutated fragments were compared with those carrying original genomic fragments, it became apparent that the integrity of TCF7L2-binding motif was significantly necessary in the binding events between TCF7L2 and PIK3R1 promoter. As shown in Fig. 6, the various degrees of luciferase activity reduction demonstrated that there was obviously different affinity of CAA in the binding events between TCF7L2 and PIK3R1 promoter. The analysis provided statistical validation to this observation and strikingly stressed the significance of TCF7L2-binding motif in the investigation of TCF7L2-mediated transcriptional regulation.

## Discussion

Growing evidence suggests that TCF7L2 (formerly TCF4), a key effector of the Wnt signaling pathway, plays a central role in directing glucose homeostasis in the pancreas and is required for maintaining GSIS and beta-cell survival [29]. Notably, recent studies have shown that TCF7L2 could activate PI3K/AKT pathway in insulin-producing beta-cells of pancreas [30], which supports the hypothesis that TCF7L2 may exert its effect on glycometabolism via the activation of PI3K/AKT signaling pathway. Given that TCF7L2 is normally deemed to be a positive regulator of pancreatic β-cell function, we made great efforts to investigate the mechanisms through which TCF7L2 exhibits its effect and strikingly identified a potential role for TCF7L2 in glycometabolism by targeting PI3K/AKT pathway.

Considering that at-risk single nucleotide polymorphisms (SNPs) rs7903146 and rs12255372 of TCF7L2 have previously been suggested to associate with maintained insulin sensitivity but defects in β-cell function and insulin release [8, 16], we provided a detailed investigation of the effects of altering TCF7L2 content on insulin secretion and on the expression of P85 which is pivotal to the activation of PI3K/AKT signaling. The present study showed that inhibition of TCF7L2 activity by KD-1 or KD-3 virus could increase the expression levels of PIK3R1 and its encoding protein PI3K p85, followed by the decreased activation of p-AKT. These findings led us to postulate that inhibition of insulin secretion may be the result of increased expression of PIK3R1 and its encoding protein p85, which directly leading to a decrease activation of PI3K/AKT signaling. To further validate this possibility, MIN6 cells treated with lenti-TCF7L2 virus were analyzed to reveal increases in p-AKT and insulin secretion but decrease in protein level of p85. These data demonstrated that a lacking of functional TCF7L2 could lead to defective GSIS, which is probably attributed to changes in the expression of PIK3R1 and activation of AKT signaling pathway. However, several previous findings suggested that the acute effects of TCF7L2 depletion on insulin secretion are the result of changes in insulin maturation but not insulin production [31], which was contradicted with our observations. The mechanisms behind these conflicting viewpoints remain obscure and future studies will be required to explore the concrete role of TCF7L2 in glycometabolism.

As TCF7L2 lies at the foot of Wnt signaling pathway and transduces signals generated by Wnt receptors to modify expression of multiple genes which are associated with proliferation and protein synthesis [32], a comprehensive identification of the specific binding sites is essential for a more complete understanding of the genome-wide TCF7L2 binding profile and its role in glucose metabolism. However, information on transcription factor binding-site specificity is often incomplete or biased by the prediction methods used. ChIP has been used to generate an exhaustive map of active complex-bound promoters in human fibroblast cells [33], which allows for the capture of the binding events between transcription factors and their candidate targets. As TCF7L2 target genes are tissue and context-specific, the investigation of DNA binding patterns of TCF7L2 across the genome indicated that TCF7L2 specifically binds to multiple genes that are important in regulation of glucose metabolism in hepatocytes, which including *Pck1*, *Fbp1*, *Irs1*, *Irs2*, *Akt2*, *Adipor1*, *Pdk4 and Cpt1a* [34]. A surprisingly short list of validated TCF7L2 cardiac-specific target genes is also indentified, such as *Hand2, Tbx20, Rock2* and *Dstn* [35]. However, the binding patterns of TCF7L2 to its candidate target genes involving in gluconeogenesis in pancreas are still unknown.

To identify the association between TCF7L2 and PIK3R1 promoter in a more comprehensive manner, we scored the fold enrichments of specific genomic fractions using ChIP-PCR in our present study. As shown in Figs. 3 and 4, the contiguous fragments of PIK3R1 promoter corresponding to specific primers 1, 2, 6, 7, 8, 9 and 10 were significantly enriched after quantitative PCR, albeit to various extents. These observations were independently replicated in the luciferase reporter system, in which luciferase plasmids were built to record the relative luciferase activity over the naked plasmids. Only two of the 8 groups enhanced the transcription of luciferase reporter in this assay, which further underscored the specificity of the TCF7L2-binding profile. It is therefore undoubted that the binding events between TCF7L2 and specific segments of PIK3R1 promoter corresponding to primers 1–2 and 6–10, and the binding strength altered upon the specific binding regions.

Identification of TCF7L2-binding regions is essential for a more complete elucidation of the molecular mechanisms by which TCF7L2 regulates the transcription of its target genes. There was a striking bias for TCF7L2-binding regions to the contiguous fragments containing the evolutionary conserved motif-TCAAAG [26]. Several previous studies have demonstrated that most TCF7L2-binding regions are located at large distance from transcription start sites and significantly correlate with Wnt-responsive gene expression profiles derived from primary human adenomas  [36]. To evaluate the potential correlation between TCF7L2-binding motif occupancy and transcriptional change of PIK3R1, which was crucial for the activation of PI3K/AKT signaling pathway, we proceeded to investigate the genomic distribution of conserved TCF7L2-binding motif in PIK3R1 promoter. The plasmids carrying CAA mutated fragments mostly destroyed the luciferase activity of TCF7L2 samples, which further stressed the importance of TCF7L2-binding motif integrity in the mediated transcriptional regulation of TCF7L2.

## Conclusion

In our present study, efforts have been made with great respect to characterizing the binding events between TCF7L2 and PIK3R1 promoter, as well as defining the effects which TCF7L2 exerts on the activation of PI3K/AKT signaling pathways and insulin secretion. Indeed, multiple genetic disorders, primarily metabolic and cardiovascular, are significantly due to an extreme upstream TCF7L2-binding element driving the transcription of metabolism-associated genes [26, 36]. However, more representative and comprehensive studies are needed to clarify the underlying genetic effects of TCF7L2.

## Data Availability

All data generated and/or analyzed during this study are available from the corresponding author upon reasonable request.

## Abbreviations

TCF7L2: transcription factor 7-like 2
T2DM: type 2 diabetes mellitus
PI3K/AKT pathway: phosphoinositide 3-kinase/protein kinase B pathway
ChIP: chromatin immunoprecipitation
PCR: polymerase chain reaction
GSIS: glucose stimulated insulin secretion
SNP: single nucleotide polymorphism
PIK3R1: phosphoinositide-3-kinase regulatory subunit 1
